# Application of Mass Spectrometry for Analysis of Nucleobases, Nucleosides and Nucleotides in Tea and Selected Herbs: A Critical Review of the Mass Spectrometric Data

**DOI:** 10.3390/foods13182959

**Published:** 2024-09-18

**Authors:** Magdalena Frańska, Rafał Frański

**Affiliations:** 1 Institute of Chemistry and Technical Electrochemistry, Poznań University of Technology, Berdychowo 4, 60-965 Poznań, Poland; 2 Faculty of Chemistry, Adam Mickiewicz University, Uniwersytetu Poznańskiego 8, 61-614 Poznań, Poland; franski@amu.edu.pl

**Keywords:** nucleobases, nucleosides, nucleotides, mass spectrometry, product ions, fragmentation pathways, tea, herbs

## Abstract

The main and most commonly known biological function of nucleobases, nucleosides, and nucleotides is usually associated with the fact that they are the building blocks of nucleic acids. However, these compounds also belong to plant secondary metabolites, although in that role they have attracted less attention than the others, e.g., terpenes, phenolics, or alkaloids. The former compounds are also important constituents of the human diet, e.g., as ingredients of tea and herbs, endowing them with specific taste qualities and pharmacological activities. Liquid chromatography–mass spectrometry seems to be the most important analytical method that permits the identification and determination of nucleobases, nucleosides, and nucleotides, along with the other metabolites. The main goal of this review is to discuss in detail the aspects of mass spectrometric detection of nucleobases, nucleosides, and nucleotides in tea and selected herbs. An important conclusion is that the identification of the compounds of interest should be performed not only on the basis of [M + H]^+^/[M − H]^−^ ions but should also be confirmed by the respective product ions; however, as discussed in detail in this review, it may sometimes be problematic. It also clear that all difficulties that may be encountered when analyzing plant material are caused by the complexity of the analyzed samples and the need to analyze different classes of compounds, and this review absolutely does not debase any of the mentioned papers.

## 1. Introduction

Nucleobases, nucleosides, and nucleotides may be regarded as compounds of primary importance and as essential for life since they are the building blocks of DNA and RNA. Nucleotides also carry out some other important biological functions, e.g., nicotinamide adenine dinucleotide (NAD^+^) and nicotinamide adenine dinucleotide phosphate (NADP^+^) are important redox coenzymes in numerous metabolic pathways [1], and adenosine triphosphate (ATP), besides its role as a coenzyme, plays a crucial role in cellular metabolic processes as a carrier of chemical energy [2]. A wide range of derivatives of nucleobases, nucleosides, and nucleotides have found pharmaceutical applications, e.g., 5-fluorouracil has been widely used for treatment of various types of cancer [3], inosine pranobex is a common antiviral drug [4], and various nucleoside/nucleotide analogues are also antiviral drugs [5]. Moreover, nucleobases, nucleosides, and nucleotides belong to the plant secondary metabolites, although they are definitely less common than the other ones e.g., terpenes, phenolics, or alkaloids, and as such they have attracted less attention than others; therefore, in review papers devoted to plant secondary metabolites, these compounds are often not mentioned [6,7,8].

In order to understand the biochemical processes, mass spectrometric analysis of nucleobases, nucleosides, and nucleotides is often necessary, and it would not be an exaggeration to claim that this holds true for all biochemical branches of sciences. Probably the first review devoted to the mass spectrometric analysis of nucleosides and nucleotides was that published in 1998 by Esmans et al., in which much attention was paid to the coupling of liquid chromatography with fast atom bombardment (FAB) and thermospray ionization, i.e., ionization methods that are no longer used [9]. Of course, the application of liquid chromatography–electrospray ionization was also discussed in the review by Esmans et al., although it was a relatively new technique in the nineties. A few years later, Banoub et al., published a review devoted to the mass spectrometric analysis of nucleosides, nucleotides, oligonucleotides, and nucleic acids, although this review was mainly devoted to the analysis of oligonucleotides and nucleic acids [10]. At the same time, Willems et al., published a review devoted to the analysis of nucleic acid constituents by capillary electrophoresis–mass spectrometry [11]. Special emphasis was given to the sample preparation, capillary electrophoresis modes, and capillary electrophoresis–mass spectrometry interfaces. Relatively recently, a comprehensive review devoted to the mass spectrometric analysis of nucleosides and nucleotides was published by Dudley and Bond, who discussed many issues in detail, e.g., fragmentation pathways, the most common mass spectrometry techniques used for their detection, DNA adduct analysis, etc. [12]. As far as the type of biological samples studied are concerned, the authors paid the most attention to the analysis of urinary nucleosides, since urinary modified nucleosides are biomarkers of cancer diseases. The most recent review was focused on the analysis of modified nucleosides (cancer biomarkers) by using isotope dilution mass spectrometry, and particular attention was paid to the application of liquid chromatographic techniques, e.g., bidimensional liquid chromatography and chemical derivatization procedures [13].

In the review papers devoted to the plant metabolites, the nucleobases, nucleosides, and nucleotides, as less common metabolites, are often disregarded [14,15,16]. On the other hand, relatively recently Straube et al., published an excellent review devoted to the analysis of nucleosides and nucleotides in plants [17], in which the state-of-the-art of the sample preparation methods was discussed in detail in six sections (disruption of the tissue, quenching of the sample, extraction of NTS and NS from plant samples, solid-phase extraction, derivatization, reduction in sample volume). The used chromatographic separation methods and mass spectrometric techniques were comprehensively described in two sections (chromatographic separation and mass spectrometry, respectively). It is also worth adding that Straube and Herde have described in detail the protocol to isolate nucleotides and nucleosides from plants and to prepare the samples containing these compounds, prior to injection to LC-MS [18].

Tea is one of the most common non-alcoholic drinks in the world; therefore, the compounds present in tea, e.g., nucleosides, are heavily consumed and may contribute to its flavor. The nucleosides present in herbs may contribute to their therapeutic properties. This review covers the mass spectrometry application for the analysis of nucleobases, nucleosides, and nucleotides in teas and selected herbs, which may be regarded as representative plant sources. The major aim of the review is a detailed discussion of the mass spectrometric detection of the title compounds. In the discussed papers, these compounds are usually called nucleotides and derivatives; therefore, this nomenclature will be also used in this review. Although a review is often critical, it has to be stressed that it definitely does not debase any of the discussed papers. This review is divided into three sections, according to the contribution of the nucleotides and their derivatives to the study. The first section covers the research focused on the analysis of nucleotides and derivatives, the second section covers the studies of plant metabolites, including a significant contribution of these compounds, and the third section describes examples of studies of plant metabolites, to which nucleotides and derivatives have brought a moderate or minor contribution. Therefore, analyses of the same sources are sometimes discussed in different sections, e.g., *Abelmoschus manihot*. Of course, we are aware that this classification may sometimes be disputable.

## 2. Principles of Mass Spectrometric Analysis of Nucleotides and Derivatives

These compounds have been known for years, and their liquid chromatography–mass spectrometric analysis should not raise any problems. In reverse-phase (RP) chromatography analysis, they have relatively short retention times due to their hydrophilic properties, and sometimes their proper chromatographic separation may cause some problems. Therefore, hydrophilic interaction liquid chromatography (HILIC) has been widely used for their analysis [19]. On the other hand, there are examples showing that reverse-phase chromatography yielded better separation of these compounds [20]. It is understood that the chromatographic separation of nucleotides and derivatives is very sensitive to the stationary and mobile phases’ properties, namely, minor changes in the properties may significantly affect their separation (peak resolution), as well as to other analytical parameters (e.g., temperature, flow rate), and sometimes minor changes in chromatographic conditions may significantly affect the chromatographic results. The chromatographic conditions, namely, type of chromatography (RP or HILIC), type of column, and solvents (mobile phases), used in the papers discussed in this review are summarized in the Supplementary Materials (Table S1).

**Figure 1:**
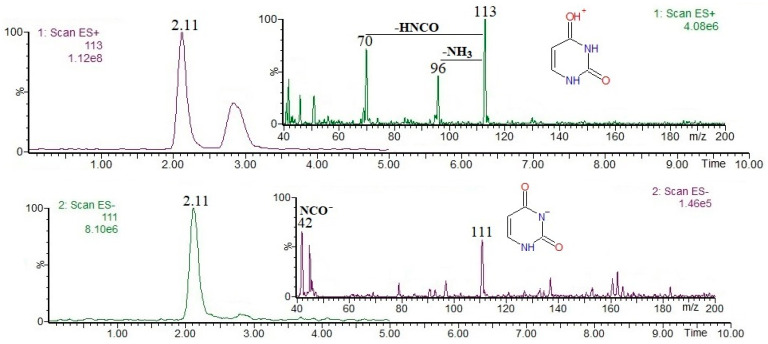
Single-ion chromatograms of uracil [M + H]^+^ and [M − H]^−^ ions, and ESI mass spectra.

**Figure 2:**
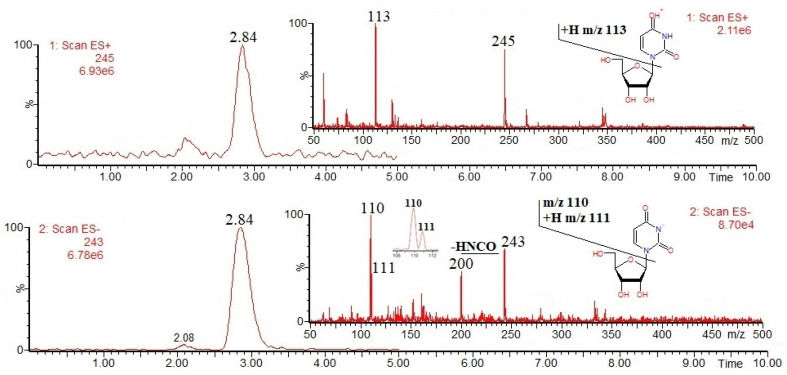
Single-ion chromatograms of uridine [M + H]^+^ and [M − H]^−^ ions, and ESI mass spectra.

**Figure 3:**
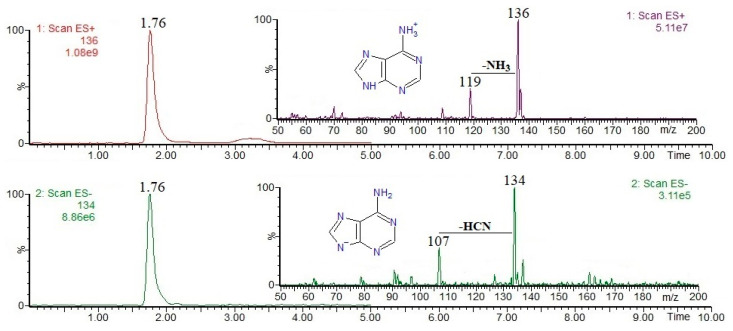
Single-ion chromatograms of adenine [M + H]^+^ and [M − H]^−^ ions, and ESI mass spectra.

**Figure 4:**
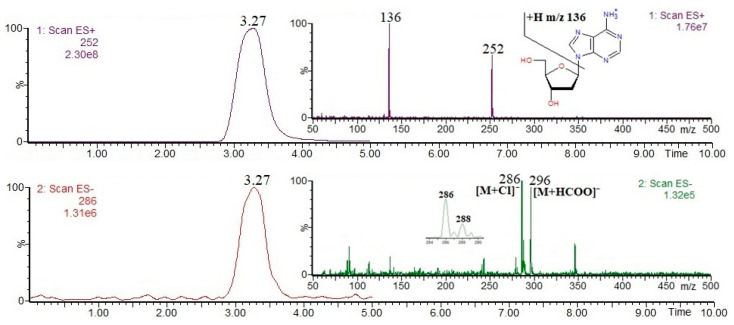
Single-ion chromatograms of adenosine [M + H]^+^ and [M − H]^−^ ions, and ESI mass spectra.

**Figure 5:**
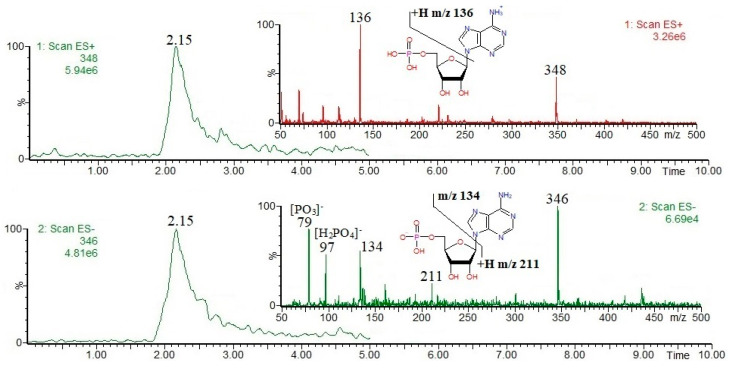
Single-ion chromatograms of AMP [M + H]^+^ and [M − H]^−^ ions, and ESI mass spectra.

Nucleotides and derivatives may be successfully analyzed by using LC-MS in both positive and negative ion modes (as [M + H]^+^ and/or [M − H]^−^ ions); obviously, it is clear that their MS(+/−) responses strongly depend on their acid/base properties. In general, mass spectrometric fragmentation pathways of [M + H]^+^ and/or [M − H]^−^ ions are well documented in the literature, and the most common nucleotides and derivatives can be easily determined on the basis of the available mass spectral databases, e.g., https://massbank.eu/MassBank/ (accessed on 1 August 2024). Figure 1, Figure 2, Figure 3, Figure 4 and Figure 5 show the results of an HPLC-MS(+/−) analysis of a mixture of five compounds as a representative example. The HPLC-MS conditions were as those described elsewhere [21] when used for the analysis of phenolic compounds in plant sources, and the nucleotides and derivatives are characterized by shorter retention times than phenolic compounds. Since adenosine has the weakest acidic properties among the analyzed compounds, under the conditions used, it did not form [M − H]^−^ ions, instead forming [M + Cl]^−^ and [M + HCOO]^−^ ions (Figure 4, chlorides are common contamination, and formic acid was used to acidify the mobile phase [21]).

The fragmentation of nucleobases consists of a loss of small molecules, e.g., NH_3_ or HCN (Figure 1 and Figure 3). The fragmentation of nucleosides involves the breaking of N-glycosidic bonds, which, accompanied by the loss of mass 132, corresponds to the loss of a ribose moiety, while the loss of mass 116 corresponds to the loss of a deoxyribose moiety (Figure 2 and Figure 4). In the positive ion mode, the formed product ions correspond to the protonated nucleobase molecules, while in the negative ion mode, to the deprotonated nucleobase molecules. However, in the negative ion mode, sometimes the homolytic breaking of N-glycosidic bonds may occur, leading to the formation of radical anions (odd-electron product ions), e.g., [uracil − 2H]^●−^ at *m*/*z* 110 (Figure 2). The fragmentation of nucleotides also consists of the breaking of N-glycosidic bonds, leading to the formation of protonated/deprotonated nucleobase molecules; apart from that, their fragmentation involves the breaking of phosphoester bonds, leading to the formation of phosphorus-containing anions, e.g., [PO_3_]^−^ and [H_2_PO_4_]^−^ at *m*/*z* 79 and 97, respectively (Figure 5). Sometimes, other fragmentation pathways may be observed, especially for nucleoside [M + H]^+^/[M − H]^−^ ions, e.g., the loss of HNCO molecule from [uridine-H]^−^ ion [22], or the formation of a ribose moiety product ion at *m*/*z* 133 from [uridine + H]^+^ ion [12]. The above briefly describes principles of fragmentation of nucleotides, and derivatives should be considered during LC-MS analysis, e.g., in order to choose the appropriate qualifier/quantifier ions for analysis in the multiple reaction monitoring (MRM) mode.

## 3. Tea- and Herb-Targeted Analysis of Nucleotides and Derivatives

The papers devoted to the LC-MS analysis of herbs or tea, in which analyses of nucleotides and derivatives were the only or the main goal, are rather limited in number. Below, a few interesting examples of such papers are discussed.

Du et al., have reported a UPLC-MS approach focused on the analysis of nucleotides and derivatives in different parts of *Abelmoschus manihot*. This is a herbaceous plant, and its extracts show the potential ability to treat various diseases [23]. For chromatographic separation, the authors used a BEH Amide column, while the mobile phases were water and acetonitrile solutions of HCOONH_4_/CH_3_COONH_4_/HCOOH. MS detection was performed in the multi-reaction monitoring (MRM) mode. The authors have successfully determined twelve nucleotides and derivatives. From a practical point of view, a problematic issue may be the poor separation of thymidine/thymine (rt = 1.19/1.21 min) and 2′-deoxyuridine/uracil (rt = 1.38/1.39 min). Thymidine and 2′-deoxyuridine give product ions at *m*/*z* 127 and 113, respectively, whereas thymine and uracil [M + H]^+^ ions have identical *m/z* values, respectively. Since the glycosidic bonds are weak, it can sometimes be difficult to avoid their breaking in-source [24]. Therefore, the peaks of the product ions of thymidine and 2′-deoxyuridine may overlap those of [M + H]^+^ ions of thymine and uracil, respectively, obstructing their analysis. Fortunately, in the samples analyzed by Du et al., the concentrations of thymine and uracil were much higher than those of thymidine and 2′-deoxyuridine, thus the obstruction, if any, was negligible. Another issue which needs to be dealt with is the lack of product ions for the MRM analysis of nucleobases. For these compounds, the authors used [M + H]^+^ → [M+H]^+^ transitions (e.g., 137 → 137 for adenine), which may not yield the desired specificity. Another disputable issue is the analysis of guanine, namely, the preparation of its stock solution. For all analytes, not excluding guanine, the authors claimed that the concentration was 1 mg/10 mL. Guanine is hardly soluble in water in and in other solvents, and reaching this concentration may be problematic [25].

Guo et al., have analyzed nucleotides and derivatives in the fruits and leaves of *Ziziphus* plants used in China as medicine and food. Twenty compounds were determined in the positive ion mode by MRM analysis. There were two pairs of compounds that were not separated: hypoxanthine/uridine (rt = 2.70/2.71 min) and 2′-deoxyinosine/xanthine (rt = 3.10/3.11 min) [26]. Fortunately, these coeluted compounds do not yield product ions of the same *m*/*z*, thus their coelution was not troublesome. More difficult may be the lack of the product ions for nucleobases, in addition to reaching the guanine concentration of 100 µg/mL in stock solution.

The nucleobases and nucleosides occurring in *Fritillaria cirrhosa* D. Don. (a medicinal herb commonly used in China as an antitussive and apophlegmatic agent) have been determined by Duan et al., by using HPLC-MS analysis [27]. The authors did not perform MS/MS experiments; however, for nucleosides, the characteristic product ions were observed, and it can be taken for granted that they were formed due to the fragmentation in-source (the loss of a ribose moiety for ribosides (loss of mass 132) and the loss of a deoxyribose moiety for deoxyribosides (loss of mass 116)). The product ions were observed for nucleobases (adenine and hypoxanthine), and for most of the detected compounds, [M + Na]^+^ ions were also observed. The MS results presented by Duan et al., do not raise any doubts, although there are a few undesirable typos in Table 1, namely, for thymidine the MW should be 242, [M+H]^+^ at *m*/*z* 243, [M+Na]^+^ at *m*/*z* 265, *m*/*z* 127 corresponds to the [M + H-deoxyribose]^+^, and for 2-deoxyadenosine the *m*/*z* 136 also corresponds to the [M + H-deoxyribose]^+^ [27].

Hydrophilic interaction chromatography coupled with ultra-high resolution mass spectrometry (HILIC-UHRMS) has been used by Zhao et al., to analyze changes in the content of nucleotides and derivatives during the white tea withering process. It was suggested that guanosine 5’-monophosphate (GMP) and adenosine 5’-monophosphate (AMP) may significantly affect the umami taste [28]. The obtained accurate masses of [M + H]^+^ ions match the theoretical ones perfectly. On the other hand, the claimed MS experimental conditions, namely, “dd-MS2”, suggested the occurrence of a fragmentation process; however, the detected product ions were not reported. It should be also pointed out that stock solutions of some nucleobases, e.g., guanine, were prepared by dissolving them in 1 M sodium hydroxide aqueous solution (guanine is not water soluble but is soluble in NaOH solution); therefore, there is no doubt concerning guanine solubility.

Analysis of the nucleotides and derivatives (also amino acids) in Chrysanthemi Flos (a medicinal plant used in China for thousands of years) has been reported by Chang et al., who found that these compounds can be used as quality markers of the geographical origin of the herb [29]. The UPLC-MS experiments were performed in chromatographic conditions similar to those used by Du et al. [23] and, analogously, the problem with the separation of nucleobases/nucleosides appeared, namely, uracil/uridine at rt = 1.82/1.81 min and guanine/2′-deoxyguanosine at rt = 3.09/3.09 min. Furthermore, similarly as in the work by Du et al., the preparation of the guanine stock solution at the concentration of 1 mg/10 mL may be questioned. In contrast to the study by Du et al., Chang et al., used the appropriate product ions for the MRM analysis of nucleobases (113 → 70 for uracil, 152 → 110 for guanine).

In another paper, Chang et al., studied the dynamic changes in nucleotides and derivatives (together with amino acids) in flowering stages of Chrysanthemi Flos. The provided precursor ions and product ions do not raise any doubts for almost all nucleosides/nucleotides, and the product ions corresponded to the protonated nucleobases [30]. Among the identified nucleosides, authors have detected a relatively rare one, namely, 2′-deoxyuridine (although its concentration was low), for which the product ion corresponded to the deoxyribose moiety ([M + H-uracil]^+^ at *m/z* 117), which is correct [31].

In the paper by Zhang et al., the nucleotides and derivatives (together with amino acids and alkaloids) present in the leaves of mulberry, a herb commonly used in traditional Chinese medicine (also the well-known only food of silkworms), were analyzed [32]. The authors optimized the experimental UPLC-MS conditions and provided all experimental details. From a practical point of view, one problematic issue may be the poor separation of 2′-deoxycytidine/2′-deoxyuridine (rt = 2.99/3.01 min, ions [M + H]^+^ at *m*/*z* 228/229) and 2′-deoxyinosine/2′-deoxyadenosine (rt = 1.66/1.68 min, ions [M + H]^+^ at *m*/*z* 253/252). When the molecular weights differ only by one unit, the isotope peaks (containing one ^13^C atom) of the lighter compounds may overlap the peaks of [M + H]^+^ ions of the heavier ones, affecting their analysis [33].

Optimization of the analytical method for the analysis of nucleotides and derivatives in different types of Yunnan large-leaf tea was the outcome of the study reported by Wang et al. [34]. The nucleobases were classified as alkaloids and discussed together with them. The other analyzed compounds were amino acids. The authors found that vortex-assisted extraction provides high efficiency. The employed method of analysis was hydrophilic interaction chromatography (HILIC), followed by triple quadrupole tandem mass spectrometry, and all experimental details were comprehensively described. All compounds, besides xanthine, were analyzed in the positive ion mode. As expected for the HILIC conditions, the nucleobases were eluted first, followed by nucleosides and then nucleotides. It may be disputable that the negative mode was applied for only xanthine (purine base), probably as the negative mode would also be appropriate for nucleotides. It has to be stressed that in order to prepare stock solutions of guanine and xanthine (which are hardly soluble in water), Wang et al., used a 0.1 M NaOH solution [34].

Using different scanning modes (parallel reaction monitoring and data-independent acquisition), Wang et al., have determined the nucleotides and derivatives (together with amino acids and alkaloids) present in Yunnan large-leaf white tea, and their work can be considered as a model study employing mass spectrometry for the analysis of these compounds in a plant source [35]. In order to generate the perfect Gaussian-like shapes of chromatographic peaks, the authors optimized various parameters, namely, selected precursor and product ions (performed faultlessly), the width time window for the scanning modes, high collision-induced dissociation (HCD) voltage, polarity switching mode, and MS resolution. The presented results may be very useful for scientists working with the UPLC–Orbitrap–MS apparatus [35].

## 4. Tea and Herb Analyses to Which Nucleotides and Derivatives Have Brought a Significant Contribution

This section covers the papers in which nucleotides and derivatives were identified together with other abundant compounds present in plants, e.g., catechins and theaflavins.

Wang et al., have detected several nucleotides and derivatives in *Semen ziziphi spinosae* decoctions (a herb commonly used in China for thousands of years to treat various ailments, e.g., insomnia, anxiety, forgettery, dreaminess, and others) [36]. The authors have performed UPLC-Q/TOF-MS/MS analysis and have demonstrated that adenosine, together with seven phenolic compounds and one alkaloid (magnoflorine), may be used as a quality marker to control the analyzed decoctions. However, the detection of nucleosides and nucleotides raises some doubts. For example, for adenosine ([M − H]^−^ parent ion) the authors claim detection of the product ions at *m*/*z* 202, 167, and 62 (nominal values), which do not seem to be characteristic values (https://massbank.eu/MassBank/) (accessed on 1 August 2024); the [M − H]^−^ ion of uridine monophosphate was reported at *m/z* 243, whereas it should be at *m*/*z* 323 (*m*/*z* 243 corresponds to the [M − H]^−^ ion of uridine), and the question is why the authors claim the detection of guanosine hydrate. Furthermore, in order to support the MS detection of nucleosides and nucleotides, the authors cited a paper by Han et al., which does not contain these data [37].

Using LC-MS, Bai et al., have analyzed changes in the contents of small-leaved Kuding tea upon heat treatment. Among the analyzed compounds, the greatest changes in content were observed for nucleotides. The authors claimed the detection of several nucleotides and related compounds, e.g., nucleotide sugars (uridine diphosphate xylose/glucose); however, the MS details of their detection (retention times, *m*/*z* values of parent and product ions) were not provided [38].

Ge et al., have compared the chemical profiles of Pu-erh teas from thousands-of-years-old trees with those of Pu-erh teas from ecological trees [39]. Several nucleobases and nucleosides were detected. The authors provided the obtained accurate masses of both parent ([M + H]^+^ ions) and product ions; however, a few of the latter are disputable. For example, it is difficult to rationalize the hypoxanthine product ions at *m*/*z* 106.0410, 78.0346, and 52.0314, since the characteristic hypoxanthine product ions should be at *m*/*z* 119, 110, and 94 [40]. For deoxyguanosine, the authors reported product ions at *m*/*z* 191.9528, 136.0614, 85.0288, and 57.0337; thus, the most characteristic product ion at *m*/*z* 151 (formally protonated guanine), formed by the loss of deoxyribose moiety (loss of mass 116), is missing.

Changes in the chemical composition of oolong tea from New Zealand upon its manufacturing have been investigated by Frasel et al. Among the compounds of key importance to distinguish pre- and post-degreening stages, there were four nucleotides, one nucleoside (guanosine) and one nucleobase (adenine). As perfectly discussed in detail by the authors, the presence of nucleotides and nucleoside was confirmed by the respective product ions, corresponding to the protonated/deprotonated nucleobases [41]. The only disputable issue is the fact that adenine was detected in the negative ion mode, whereas adenosine monophosphate (AMP) was found in the positive ion mode, since the opposite situation would be expected (adenine is more prone to protonation than deprotonation, and AMP the other way round).

The effect of storage time (aging process) on metabolome changes in oolong teas has been of interest for Hong et al. Several nucleotides and their derivatives were found to be so-called differential metabolites. For most of them, the mass spectrometric detection does not raise any doubts, with the exception of uracil, for which the claimed product ion at *m*/*z* 80(−) is not characteristic, and also of 5,6-dihydro-5-methyluracil (5,6-dihydrothymine) and 5-aminoimidazole ribonucleotide, for which it is difficult to rationalize the product ions at *m*/*z* 84(+) and 104(+), respectively [42]. 5,6-Dihydro-5-methyluracil should yield a product ion at *m*/*z* 86(+) (loss of mass 43) [43], and 5-aminoimidazol ribonucleotide should yield product ions at *m*/*z* 162, 260, 278 [44]. For 6-methylmercaptopurine, the authors claim that the detection of a product ion at *m*/*z* 121, which is a low abundance in the product ion mass spectrum of this compound (https://massbank.eu/MassBank/) (accessed on 1 August 2024); for this compound, the appropriate product ion should be at *m*/*z* 126 [45].

Changes in the contents of metabolites in Liupao tea that take place during the tank fermentation process have been analyzed by Long et al. The metabolites were detected on the basis of comparison of the mass spectra obtained with those present in the mzcloud database [46]. Among the forty metabolites of importance, there were seven nucleotides and derivatives, and their contents significantly increased during the fermentation process, in contrast to the contents of the other components. Among the nucleotides and derivatives, the authors claimed the detection of a relatively rare nucleoside, namely, cytarabine (cytosine arabinoside). Since it is the isomer of cytidine, it would be desirable to show more experimental results that clearly indicate that these compounds were not missed. A similar study was reported Wang et al., who scrutinized changes in the contents of metabolites in Liupao tea during tank and traditional fermentation processes [47]. Analogously, the metabolites were detected on the basis of comparison of the mass spectra obtained with those present in the mzcloud database. Wang et al., found that one of the sulfur-containing nucleotides, namely, 5′-S-methyl-5′-thioadenosine, can be used for distinction of fermented tea from unfermented tea (it was absent in the fermented tea) [47].

The influence of the duration of fermentation on the taste quality and contents of metabolites of black tea was examined by Dong et al. Among the compounds found to be of importance for tea properties, the authors established that guanine, among others, may enhance the bitter flavor of black tea, and that cyclic-3′,5′-adenine nucleotide (the authors probably mean adenosine 3’,5’-cyclic monophosphate) may enhance the sweetness and reduce the freshness of black tea [48]. Although the authors used UPLC-MS/MS to analyze changes in the contents of metabolites, no important experimental details were provided, e.g., the preparation of guanine standard solution, the ions used for MRM transitions, retention times of the identified compounds, etc.

Another interesting point was the effect of drying temperature on the sensory attributes of black tea, as considered by Hua et al. The authors found that, among others, six nucleobases may significantly contribute to the taste, namely, uracil, guanine, cytosine, 5-methylcytosine, thymine, and 1-methyladenine [49]. The nucleobases were analyzed in the positive ion mode (as [M + H]^+^ ions), except for uracil, which was analyzed in the negative ion mode (as [M − H]^−^ ion at *m*/*z* 111). For most of the nucleobases, the provided product ions do not raise any doubts, except for uracil, whose claimed product ion was at *m/z* 80.

Shu et al., treated Huangjinya tea with different-colored shading nets and established that red shading nets had the most profound positive effect on tea growth. The results of their UPLC-MS/MS analyses show that the so-called top 10 upregulated metabolites include guanine, isoguanine, and 8-azaguanine [50]. However, no experimental details were provided by the authors, and it would be interesting to show how these three similar compounds have been distinguished under the used UPLC-MS/MS conditions.

The metabolomic changes in tea shoots from *Camellia sinensis* cv. Tieguanyin subjected to the sequential manufacturing processes have been considered by Li et al. Although the main analyzed metabolites were catechins, xanthine alkaloids, and amino acids, the authors also noted the effect of four nucleotides (guanosine 5′-phosphate, adenosine 5′-monophosphate, inosine 5′-monophosphate, uridine 5′-monophosphate) on the tea flavor; namely, these compounds enhanced the umami taste [51]. Three nucleotides were detected in the positive ion mode (GMP, AMP, IMP) and the respective product ions corresponded to the protonated nucleobases, with the exception of uridine 5′-monophosphate detected in the negative ion mode, whose product ion was formed by the uracil loss. Interestingly, in the negative ion mode, the authors also detected adenosine 2′-phosphate (isomer of adenosine 5′-monophosphate) [51]. Similar results have been reported by Xue et al., who analyzed the impact of different fixation methods on the sensory quality and metabolite contents of green tea. Xue et al., disclosed the influence of the same nucleotides on the umami taste and identified two isomers of adenosine 5′-monophosphate, namely, adenosine 2′-monophosphate and adenosine 3′-monophosphate (3’-adenylic acid) [52]. Adenosine 5′-monophosphate was detected in the positive ion mode, whereas its isomers were found in the negative ion mode. These results may suggest different mass spectrometric behaviors in adenosine monophosphate isomers, namely, adenosine 5′-monophosphate yields better signal in the positive ion mode, whereas adenosine 2′-monophosphate and adenosine 3′-monophosphate yield better signal in the negative ion mode.

Xue et al., focused on the metabolite conversion that occurs during the manufacturing process of shaken black tea. Cytidine 5ʹ-monophosphate (CMP), cytidine 5’-diphosphate (CDP), and cytidine 2’,3’-cyclic phosphate (2’,3’-cyclic CMP) [53] were among the main taste-determining compounds whose contents changed during the process. CMP was detected in the positive ion mode at *m*/*z* 306, which is disputable as [CMP + H]^+^ should be at *m*/*z* 324, and [CMP+H]^+^ does not lose a water molecule. CDP was detected in the negative ion mode at *m*/*z* 384, which is also disputable as [CDP − H]^−^ should be at *m*/*z* 402 and [CDP-H]^−^ also does not lose a water molecule. 2’,3’-Cyclic CMP was detected in the negative ion mode at *m*/*z* 402, which is correct. It should be added that the reported product ions for all these three compounds (e.g., at *m*/*z* 112 for CMP, at *m*/*z* 79 for CDP and 2’,3’-cyclic CMP) are also correct (https://massbank.eu/MassBank/) (accessed on 1 August 2024).

The conversions of metabolites of white peony teas related to storage sensory changes were a subject of interest for Fan et al. A few nucleotide derivatives were found to belong to the so-called key metabolites. The claimed precursor/product ions are correct, although the product ion at *m*/*z* 121 claimed for 6-methylmercaptopurine may be disputable, as mentioned above [54]. Among the key nucleotide derivatives, there was a relatively rare nucleoside, namely, 9-(β-D-arabinofuranosyl)hypoxanthine. As it is an isomer of a common nucleoside inosine, it would be desirable to show the LC-MS/MS differentiation of these isomers.

Changes in the chemical composition of Fu brick tea that take place during the fermentation process have been described by Jia et al., and the characteristic components included guanine. Its stock solution (1 g/L) was prepared in methanol with an addition 1 M NaOH; therefore, there is no doubt concerning the guanine solubility [55]. On the other hand, the peaks of the product ions at *m*/*z* 122, 121, 120, and 93, as shown in the mass spectrum provided by the authors, are difficult to rationalize, since the characteristic product ions derived from guanine [M + H]^+^ ion should be at *m*/*z* 135, 110, and 82 [56,57].

Jon et al., analyzed *Abelmoschus manihot* flower extracts. This is a herbaceous plant whose extracts show a potential ability to treat various diseases. The identified compounds included a few nucleobases and nucleosides, among them guanine and adenosine or 2′-deoxyguanosine [58]. For guanine, the authors claim to have found the product ion at *m*/*z* 93, which is incorrect (https://massbank.eu/MassBank/) (accessed on 1 August 2024). The authors did not differentiate between adenosine and 2′-deoxyguanosine, rightly arguing that these are isobaric compounds. On the other hand, these compounds should be differentiated on the basis of the characteristic product ions, namely, at *m*/*z* 136 for adenosine and at *m*/*z* 152 for 2′-deoxyguanosine. As the authors found a product ion at *m*/*z* 136, the detected compound was most probably adenosine. 

## 5. Other Examples of Analyses of Nucleotides and Derivatives in Tea and Herbs

This section covers examples of the studied plant metabolites to which the nucleotides and derivatives had brought moderate or minor contribution. However, even in such cases, they may be of importance with respect to the health-promoting or taste properties of herbs and tea. Therefore, even if the nucleotides and derivatives are detected in the process of identification of the main ingredients, their detection should be correctly justified.

Fu et al., have detected a few nucleobases and nucleosides in a decoction of Banxia Baizhu Tianma (Chinese herbal formula for hypertension). The authors observed, characteristic for nucleosides, loss of a ribose moiety (loss of mass 132), followed by the loss of ammonia (loss of mass 17), and the latter process, as expected, was observed for nucleobases [59]. The authors identified a relatively rare but interesting nucleoside, namely, N6-(4-hydroxybenzyl)-adenosine, which may be used for treating alcohol use disorder [60]. The reported product ions at *m*/*z* 242 and 136 perfectly match those reported elsewhere [61].

The determination of bioactive constituents in different parts of *Tetrastigma hemsleyanum* (its roots are used in traditional Chinese medicine, and its stems and leaves are consumed as functional tea and food supplementation) was the aim of Luo et al. [62]. A few nucleosides and nucleobases were detected, although their percentage contents were very low in comparison to those of polyphenols and amino acids. The presented mass spectrometric results do not raise any doubts, although there is a typo, namely, the parent/product ions of guanine should be at *m*/*z* 152/135 (not 153/136). More problematic is the claimed guanine concentration in the reference standard solutions (prepared in water), namely, 25 μg/mL [62]. As mentioned earlier, reaching this concentration would be problematic [25].

The metabolites of *Dendrobium catenatum* leaves (Chinese medicinal herbs) subjected to different types of drying processes have been reported by Liu et al. The authors identified two nucleotides, namely, cytidylic acid (CMP) and uridylic acid (UMP), and found that the content of the CMP increased during hot air-drying and rolling-before-drying treatments [63]. The nucleotides were detected on the basis of the *m*/*z* of [M + H]^+^ ions; however, these compounds should also be detected in the negative ion mode (flavonoids were detected in both positive and negative ion modes), and the characteristic product ions formed due the cleavage of relatively week N-glycosidic/phosphoester bonds should be observed.

The chemical profile of the leaves of *Camellia nitidissima* (a kind of caffeine-less tea) has been solved by Ye et al. Among the identified compounds, the authors found adenine, guanosine, and cordycepin (3’-deoxyadenosine), and each identification was perfectly performed on the basis of the characteristic fragmentation pathways of [M + H]^+^ ions and by comparison with the standards [64]. The comparison was of crucial importance for cordycepin identification, which could be confused with its common isomer 2′-deoxyadenosine [65].

The major bioactive compounds of *Gastrodia elata* (a common herb often used in traditional Chinese medicine) were the subject of the study by Chen et al. Among the identified compounds, the authors detected two nucleosides, adenosine and uridine [66]. The detection of the former does not raise any doubts; however, for the latter, the authors claim the loss of mass 132 from the [M + Na]^+^ ion (*m*/*z* 267) and the formation of a product ion at *m*/*z* 113. This process should yield a product ion at *m/z* 135, or a product ion at *m*/*z* 113 should be formed from the [M + H]^+^ ion (*m*/*z* 245). There is also the question why in the negative ion mode the authors did not detect any product ions from uridine [M − H]^−^ ion (*m*/*z* 243) in the negative ion mode.

The chemical contents of ancient bud black tea were analyzed by Li et al., and compared with those of traditional black tea. The authors also checked the chemical changes in ancient bud black tea during fermentation [67]. Among the vast number of phenolic compounds, the authors detected only two nucleobases and three nucleosides. However, the reported MS/MS data raise some doubts, e.g., the presence of an abundant ion (100% ri) at *m*/*z* 138 for adenine, or a lack of the product ion at *m*/*z* 152 for guanosine [67].

Tan et al., have reported changes in the metabolome content of the *Camellia sinensis* L. tea leaves upon the fermentation process. The authors identified one nucleobase and four nucleosides, including two sulfur-containing ones, namely, 5′-methylthioadenosine and (S)-5’-deoxy-5’-(methylsulfinyl)adenosine, identified on the basis of the obtained accurate masses and metabolomics databases [68]. Although there is no reason to question their identification, a confirmation using the respective product ions would be desirable.

The metabolite profile of *Camellia sinensis* tea leaves under shading treatments were a subject of interest for Yang et al. Nucleotides and derivatives brought minor contributions to the detected metabolites and were unchanged, or their contents were lowered in the dark-treated samples compared to the controls [69]. No MS/MS details were provided.

The chemical composition of a Gualou Xiebai Banxia decoction, a classical traditional Chinese medicine formula for the treatment of coronary heart disease, was determined by Xiang et al. Among the 49 identified chemical constituents were two nucleosides, adenosine (which was claimed to be effective for the treatment of coronary heart disease) and uridine [70]. Identification of the latter raises some doubts, namely, as it is difficult to justify the product ions at *m*/*z* 113.9624 and 132.0997. Protonated uridine should yield a base product ion corresponding to protonated uracil (C_4_H_5_N_2_O_2_, exact *m*/*z* 113.0347). Protonated uridine may also yield a low abundant product ion corresponding to the ribose moiety (C_5_H_9_O_4_, exact *m*/*z* 133.0496) [12]. The authors identified an adenosine metabolite, described as -CH_2_ + O_2,_ in the urine and feces of rats. However, it is difficult to justify the product ion at *m*/*z* 137.0931 detected for this metabolite [70].

The chemical composition of a Yihuang decoction, the formula of a traditional Chinese medicine commonly used for the treatment of various diseases, was a subject of interest for Zhou et al. Among the 90 identified compounds, there were four nucleobases and four respective nucleosides [71]. The provided MS/MS and comprehensive discussion can be regarded as a model for the identification of nucleobases and nucleosides.

There are several reports whose results are not supported with satisfactory experimental details. Cui et al., analyzed the flavor-determining compounds of Xihu Longjing tea processed using machine and manual techniques. The identified compounds included three nucleotides and derivatives detected by UPLC-MS/MS (adenine, 5’-methylthioadenosine, uridine diphosphate glucose); however, no MS/MS details were provided [72]. Sun et al., studied variations in the chemical constituents and taste of yellow tea throughout the yellowing times. Although the content of nucleotides and derivatives in the total compounds was rather minor (7%), some of them were found to contribute to the taste (bitterness or umami). However, no *m*/*z* values of the precursor/product ions used for MRM analysis were provided [73]. An attractive area of research undertaken by Liu et al., concerned the physiological and molecular mechanisms of the adaptation of tea (*Camellia sinensis* var. Tieguanyin) to low-phosphorus environments in acidic soil. Among the compounds detected, there were eleven nucleotides in the roots. However, only the *m/z* values of [M + H]^+^ or [M − H]^−^ ions were provided. The gradient program used was not specified, no retention times and product ions were provided [74]. Deng et al., were concerned with changes in the contents of Pu-erh metabolites upon Ganpu tea processing. Nucleotides and derivatives were found to occur in low numbers among the metabolite contents (5.1%), and the authors detected only two, namely, adenosine 3’,5’-cyclic monophosphate and 5’-deoxy-5’-(methylthio)adenosine [75]. Important UPLC–ESI-MS/MS experimental parameters were provided (retention times, precursor ions, collision energies), except for the product ions, which are of crucial importance.

The chemical constituents of instant dark teas, fermented using three fungi from the *Aspergillus* genus, were determined by Liao et al. Several nucleotides and derivatives were tentatively identified, although none of them belonged to the so-called key metabolites [76]. The claimed tentative identifications of nucleobases and derivatives raise some doubts (e.g., detection of adenosine 5’-monophosphate disodium salt, guanosine 5’-monophosphate disodium salt hydrate, guanosine, and D-guanosine). No MS/MS details were provided.

The metabolomics of white tea during the hot air-drying process was analyzed by Xie et al. On the so-called list of differentially accumulated metabolites, the authors mentioned four compounds classified as nucleotides and derivatives (uracil, N6-methyladenosine, 1-methylxanthine, and 1,7-dimethylxanthine) [77]. No MS/MS data were provided, and these would be desirable to better justify the detection of a relatively rare nucleoside, namely, N6-methyladenosine. Furthermore, methylxanthines are purine alkaloids and—although they originate from nucleotides—should rather be classified as such [78].

The influence of storage time on the non-volatile constituents of two brands of Liupao tea, namely, Maosheng and Tianyu, was investigated by Huang et al. Among the 154 differential metabolites of Maosheng, there were 6 nucleotides and derivatives, and the 119 differential metabolites of Tianyu also included 6 nucleotides and derivatives [79]. The metabolites were identified on the basis of the obtained accurate masses of [M + H]^+^ ions. It would be desirable to confirm their identification, even tentatively, by determination of respective product ions, especially for relatively rare nucleosides, i.e., isopentenyladenosine or 2′-O-methyladenosine [79].

The critical compounds determining the flavor characteristics of Dianhong Congou black tea were the subject of study by Zhang et al. Among the identified compounds there was only one nucleotide, namely, uridine 5’-monophosphate (UMP), and its high content was related to the umami and sourness flavors [80]. The accurate *m*/*z* of [UMP − H]^−^ ion provided at 323.0286 perfectly matches the exact *m/z* of this ion. On the other hand, this compound was identified on the basis of the product ions at *m*/*z* 79, 173, 193, 211, 279, and 305. Those at *m*/*z* 79 and 211 are correct, whereas the others are disputable. The other ones should be at *m*/*z* 97 and 111, thus H_2_PO_4_^−^ and [U − H]^−^, respectively (https://massbank.eu/MassBank/) (accessed on 1 August 2024).

The chemical constituents of different parts of *Abelmoschus manihot* (a common medicinal plant) were a subject of interest for Yin et al. Among the 35 identified constituents, there were three nucleosides (guanosine, adenosine, inosine), and their content was relatively low in comparison to those of flavonoids and amino acids [81]. All important MS/MS data were provided and the nucleoside detection does not raise any doubts.

Variations in the contents of the metabolites of JiuKeng green tea leaves during their processing were investigated by Wang et al. The authors detected 48 nucleotides and derivatives (although their contribution to all compounds was moderate). Their work can be considered a model study with respect to the experimental details supporting the detection of these compounds [82]. For most of the nucleobases, the product ions corresponded to the loss of mass 17 (NH_3_) from [M + H]^+^ ions; for most of the nucleosides, to the loss of masses 132/116 (ribose/deoxyribose moiety) from [M + H]^+^ ions; for the nucleoside monophosphates, the product ions corresponded to the protonated or deprotonated nucleobases, and for nucleoside diphosphates, to the product ion [HP_2_O_6_]^−^ at *m*/*z* 159. It is worth adding that for uridine and methyl uridine, the authors properly observed the loss of mass 133 from [M − H]^−^ ions (homolytic N-glycosidic bond cleavage). The only disputable issues are the product ions observed for the [M − H]^−^ of uracil and xanthine, at *m/z* 80 and 151, respectively. These product ions should be at *m*/*z* 42/67 and 108, respectively (https://massbank.eu/MassBank/) (accessed on 1 August 2024).

## 6. Conclusions

Nucleobases, nucleosides, and nucleotides are important ingredients in tea and herbs, as proved by a large number of LC-MS analyses. Although their contribution to the full metabolome is usually moderate or minor, they are often of importance and responsible for specific taste qualities or pharmacological activities. Apart from the detection of [M + H]^+^ or [M − H]^−^ ions, the identification of appropriate product ions is also desirable; however, this may sometimes be problematic. Therefore, it is recommended, or maybe it should be mandatory, to use appropriate standards whenever available, to use high-resolution mass spectrometry whenever possible, and/or to perform tandem mass spectrometric experiments. Of course, the critical remarks mentioned in this review absolutely do not debase any of the mentioned papers. Our intention was to show that mass spectrometric detection should be performed with particular care to obtain results that are as reliable as possible. It is also clear that all the difficulties that may be encountered when analyzing plant material (e.g., tea and herbs) are caused by the complexity of the analyzed samples and the need to analyze different classes of compounds.

## Data Availability

The original contributions presented in the study are included in the article, further inquiries can be directed to the corresponding author.

## Supplementary Materials

The following supporting information can be downloaded at https://www.mdpi.com/article/10.3390/foods13182959/s1, Table S1: The chromatographic conditions, namely, type of chromatography (RP or HILIC), type of column, solvents (mobile phases) used in the papers discussed in this review.
