# Supplementary material for: Application of Mass Spectrometry for Analysis of Nucleobases, Nucleosides and Nucleotides in Tea and Selected Herbs: A Critical Review of the Mass Spectrometric Data
Source: Foods. 2024 Sep 18;13(18):2959. doi: 10.3390/foods13182959 (PMC11431637; doi:10.3390/foods13182959)
Supplement: Supplementary file 1 [file foods-13-02959-s001.zip › foods-3212249-supplementary.pdf]

Application of mass spectrometry for analysis of nucleobases, nucleosides and nucleotides in tea and selected herbs: A critical review of the mass spectrometric data

Magdalena Frańska [1,*] and Rafał Frański [2]

[1] - Institute of Chemistry and Technical Electrochemistry, Poznań University of Technology, Berdychowo 4, 60-965 Poznań, Poland

[2] - Faculty of Chemistry, Adam Mickiewicz University, Uniwersytetu Poznańskiego 8, 61-614 Poznań, Poland;

Table S1. The chromatographic conditions, namely type of chromatography (RP or HILIC), type of column, solvents (mobile phases) used in the papers discussed in this review

Reference	No.	Type of chromatography, column, solvents
Du et al.<br>10.1016/j.jchromb.2015.10.021	[23]	HILIC chromatography<br>ACQUITY UPLC BEH Amide column<br>Solvent A – water with 5 mM ammonium formate, ammonium acetate and 0.15% formic acid<br>Solvent B – acetonitrile with 1 mM ammonium formate, ammonium acetate solution, and 0.15% formic acid
Guo et al.<br>10.1016/j.chroma.2013.05.074	[26]	HILIC chromatography<br>ACQUITY UPLC BEH Amide column,<br>Solvent A – water with 10 mM ammonium acetate and 0.8% acetic acid<br>Solvent B – acetonitrile with 0.1% acetic acid
Duan B. et al.<br>10.1080/00032719.2011.551856	[27]	RP chromatography<br>Agilent Zorbax SB–Aq C18 column<br>Solvent A – water<br>Solvent B – methanol
Zhao et al.<br>10.1016/j.foodchem.2018.06.030	[28]	HILIC chromatography<br>Acquity UPLC BEH Amide column<br>Solvent A – water with 0.2% formic acid<br>Solvent B – acetonitrile

Chang et al.<br>10.3389/fchem.2021.689254	[29]	HILIC chromatography<br>Acquity UPLC BEH Amide column<br>Solvent A – water with 5 mM ammonium formate, 5 mM ammonium acetate and 0.2% formic acid<br>Solvent B – acetonitrile with 1 mM ammonium formate, 1 mM ammonium acetate and 0.2% formic acid
Chang et al.<br>10.1016/j.microc.2019.104500	[30]	HILIC chromatography<br>Acquity BEH amide column<br>Solvent A – water with 5 mM ammonium formate, 5 mM ammonium acetate and 0.2% formic acid<br>Solvent B – acetonitrile with 1 mM ammonium formate, 1 mM ammonium acetate and 0.2% formic acid
Zhang et al.<br>10.1002/jssc.201301267	[32]	HILIC chromatography<br>Acquity UPLC BEH Amide<br>Solvent A – water with 5 mM ammonium formate, 5 mM ammonium acetate and 0.2% formic acid<br>Solvent B – acetonitrile with 1 mM ammonium formate, 1 mM ammonium acetate and 0.2% formic acid
Wang et al.<br>10.1016/j.foodchem.2021.131201	[34]	HILIC chromatography<br>Thermo Scientific Syncronis HILIC column<br>Solvent A – water with 5 mM ammonium formate and 0.2% formic acid<br>Solvent B – acetonitrile–water (90:10, v/v) with 5 mM ammonium formate and 0.2% formic acid
Wang et al.<br>10.1016/j.chroma.2023.464601	[35]	HILIC chromatography

		Thermo Scientific Syncronis HILIC column<br>Solvent A – water with 5 mM ammonium formate and 0.2% formic acid<br>Solvent B – acetonitrile–water (90:10, v/v) with 5 mM ammonium formate and 0.2% formic acid
Wang et al.<br>10.1016/j.jpha.2019.01.001	[36]	RP chromatography<br>Waters Cortecss T3 column<br>Solvent A – water with 0.1% formic acid<br>Solvent B – acetonitrile with 0.1% formic acid
Bai et al.<br>10.1111/1750–3841.16555.	[38]	RP chromatography<br>ACQUITY UPLC® HSS T3 Waters column<br>Solvent A – water with 5mM ammonium formate<br>Solvent B – acetonitrile<br>Solvent C – water with 0.1% formic acid<br>Solvent D – acetonitrile with 0.1% formic acid
Ge et al.<br>10.1016/j.foodchem.2021.129602	[39]	RP chromatography<br>Waters ACQUITY UPLC HSS T3 column<br>Solvent A – water with 0.1% formic acid<br>Solvent B – acetonitrile with 0.1% formic acid.
Fraser et al.<br>10.1016/j.foodchem.2013.11.054	[41]	RP chromatography<br>Agilent RRHD SB–C18 column<br>Solvent A – water with 0.1% formic acid<br>Solvent B – acetonitrile with 0.1% formic acid<br><br>HILIC chromatography<br>Merck polymeric bead based ZIC–pHILIC column<br>Solvent A – water with 16 mM ammonium formate<br>Solvent B – acetonitrile with 0.1% formic acid
Hong et al.<br>10.3390/molecules26237278	[42]	RP chromatography<br>Agilent SB–C18 column<br>Solvent A – water with 0.1% formic acid

		Solvent B – acetonitrile
Long et al.<br>10.1016/j.lwt.2024.116449	[46]	RP chromatography<br>ThermoFisher Scientific, Hypersil Gold C18 column<br>Solvent A – water with 0.1% formic acid<br>Solvent B – methanol
Wang et al.<br>10.1016/j.foodchem.2022.134773	[47]	RP chromatography<br>ThermoFisher Scientific, Hypersil Gold C18 column<br>Solvent A – water with 0.1% formic acid<br>Solvent B – methanol
Dong et al.<br>10.1016/j.foodchem.2024.138680	[48]	RP chromatography<br>Agilent SB–C18 column<br>Solvent A – water with 0.1 % formic acid<br>Solvent B – acetonitrile with 0.1 % formic acid
Hua et al.<br>10.1016/j.foodchem.2023.138154	[49]	RP chromatography<br>Agilent SB–C18 column<br>Solvent A – water with 0.1 % formic acid<br>Solvent B – acetonitrile
Shu et al.<br>10.3389/fgene.2024.1365243	[50]	RP chromatography<br>Agilent SB–C18 column<br>Solvent A – water with 0.1 % formic acid<br>Solvent B – acetonitrile with 0.1 % formic acid
Li et al.<br>10.1016/j.fochx.2023.100952	[51]	HILIC chromatography<br>ACQUITY BEH Amide column<br>Solvent A – water with 2 mmol/L ammonium acetate and 0.04 % formic acid<br>Solvent B – acetonitrile with 2 mmol/L ammonium acetate and 0.04 % formic acid
Xue et al.<br>10.1016/j.fochx.2023.100943	[52]	RP chromatography<br>Agilent SB–C18 column<br>Solvent A – water with 0.1 % formic acid<br>Solvent B – acetonitrile with 0.1 % formic acid

Xue et al.<br>10.1016/j.lwt.2021.113010	[53]	HILIC chromatography<br>ACQUITY UPLC BEH Amide column<br>Solvent A – water with 25 mM ammonium acetate and 25 mM ammonium hydroxide<br>Solvent B – acetonitrile
Fan et al.<br>10.1016/j.foodchem.2021.130257	[54]	RP chromatography<br>ACQUITY UPLC HSS T3 C18 column<br>Solvent A – water with 0.04% acetic acid<br>Solvent B – acetonitrile with 0.04% acetic acid
Jia et al.<br>10.1016/j.chroma.2020.460900	[55]	HILIC chromatography<br>UHPLC Hypersil Gold HILIC column<br>Solvent A – water with 4 mM ammonium formate and 0.1% formic acid<br>Solvent B – acetonitrile with 8 mM ammonium formate and 0.1% formic acid
Jon et al.<br>10.1039/d1an02130c	[58]	RP chromatography<br>ZORBAX Eclipse Plus C18 column<br>Solvent A – water with 0.1 % formic acid<br>Solvent B – acetonitrile with 0.1 % formic acid
Fu et al.<br>10.1016/j.jpba.2019.06.030	[59]	RP chromatography<br>ACQUITY UPLC BEH C18 column<br>Solvent A – water with 0.15 % formic acid<br>Solvent B – methanol
Luo et al.<br>10.1002/pca.3127	[62]	RP Chromatography<br>BEH Shield RP C18 column<br>Solvent A – water with 0.15 % formic acid<br>Solvent B – acetonitrile
Liu et al.<br>10.3390/metabo11060351	[63]	RP Chromatography<br>Venusil MP C18 column<br>Solvent A – water with 0.5 % acetic acid<br>Solvent B – acetonitrile
Ye et al.<br>10.1002/bmc.4817	[64]	RP chromatography<br>Phenomenex Kinetex C18 column

		Solvent A – water with 0.1 % formic acid<br>Solvent B – acetonitrile
Chen et al.<br>10.1371/journal.pone.0150647	[66]	RP chromatography<br>Accucore C18 column<br>Solvent A – water with 0.1 % formic acid<br>Solvent B – acetonitrile
Li et al.<br>10.1007/s00217–022–03972–6	[67]	RP chromatography<br>ACQUITY HSS C18<br>Solvent A – water with 0.1 % formic acid<br>Solvent B – acetonitrile
Tan et al.<br>10.1016/j.foodres.2015.11.018	[68]	RP chromatography<br>Zorbax Eclipse Plus C18 column<br>Solvent A – water with mM ammonium acetate and 0.1% formic acid<br>Solvent B – methanol with 5 mM ammonium acetate and 0.1% formic acid.
Yang et al.<br>10.1016/j.foodchem.2012.07.066	[69]	RP chromatography<br>ACQUITY UPLC HSS T3 column<br>Solvent A – water with 0.1 % formic acid<br>Solvent B – acetonitrile with 0.1 % formic acid
Xiang et al.<br>10.1016/j.jep.2022.114994	[70]	RP chromatography<br>ACQUITY UPLC HSS T3 column<br>Solvent A – water with 0.1 % formic acid<br>Solvent B – acetonitrile with 0.1 % formic acid
Zhou et al.<br>10.1016/j.jpba.2022.115199	[71]	RP chromatography<br>Prazis Absolute AQ–C18 column<br>Solvent A – water with 0.3 % formic acid<br>Solvent B – acetonitrile
Cui et al.<br>10.3390/foods12244486	[72]	RP chromatography<br>ACQUITY UPLC HSS T3 column<br>Solvent A – water with 0.1% formic acid<br>Solvent B – water/acetonitrile (2/3, v/v) with 0.1% formic acid

Sun et al.<br>10.1016/j.fochx.2024.101342	[73]	RP chromatography<br>Waters ACQUITY UPLC HSS T3 C18<br>Solvent A – water with 0.04% acetic acid<br>Solvent B – acetonitrile with 0.04% acetic acid
Liu et al.<br>10.3390/ijms241512431	[74]	RP chromatography<br>Agilent SB–C18 column<br>Solvent A – water with 0.1 % formic acid<br>Solvent B – acetonitrile with 0.1 % formic acid
Deng et al.<br>10.1016/j.fochx.2023.100774	[75]	RP chromatography<br>Agilent SB–C18 column<br>Solvent A – water with 0.1 % formic acid<br>Solvent B – acetonitrile with 0.1 % formic acid
Liao et al.<br>10.3389/fmicb.2023.1124546.	[76]	HILIC chromatography<br>UPLC BEH Amide column<br>Solvent A – water with 25 mM ammonium acetate and 25 mM ammonium hydroxide<br>Solvent B – acetonitrile.
Xie et al.<br>10.1080/10942912.2023.2254948	[77]	RP chromatography<br>Acquity UPLC HSS T3 C18<br>Solvent A – water with 0.04% acetic acid<br>Solvent B – acetonitrile with 0.04% acetic acid
Huang et al.<br>10.1016/j.foodres.2023.113615	[79]	RP chromatography<br>Waters Xselect HSS T3 C18<br>Positive ion mode:<br>Solvent A – water with 0.1% formic acid<br>Solvent B – acetonitrile<br>Negative ion mode:<br>Solvent A – water with 5 mM ammonium formate<br>Solvent B – acetonitrile
Zhang et al.<br>10.3390/metabo13070864	[80]	RP chromatography<br>ACQUITY UPLC HSS T3 column<br>Solvent A – water with 0.1 % formic acid

		Solvent B – acetonitrile with 0.1 % formic acid
Yin et al.<br>10.3390/molecules26071864	[81]	RP chromatography<br>XBridge®C18 column<br>Solvent A – water with 0.1 % formic acid<br>Solvent B – methanol/acetonitrile (1/1)
Wang et al.<br>10.1016/j.foodchem.2021.130131	[82]	RP chromatography<br>Agilent SB–C18 column<br>Solvent A – water with 0.1 % formic acid<br>Solvent B – acetonitrile

23. Du, L.Y.; Qian, D.W.; Jiang, S.; Shang, E.X.; Guo, J.M.; Liu, P.; Su, S.L.; Duan, J.A.; Zhao, M. Comparative characterization of nucleotides, nucleosides and nucleobases in *Abelmoschus manihot* roots, stems, leaves and flowers during different growth periods by UPLC-TQ-MS/MS. *J. Chromatogr. B* **2015**, *1006*, 130–137.

26. Guo, S.; Duan, J.A.; Qian, D.; Wang, H.; Tang, Y.; Qian, Y.; Wu, D.; Su, S.; Shang, E. Hydrophilic interaction ultra-high performance liquid chromatography coupled with triple quadrupole mass spectrometry for determination of nucleotides, nucleosides and nucleobases in *Ziziphus* plants. *J. Chromatogr. A* **2013**, *1301*, 147–155.

27. Duan, B.; Wang, L.; Dai, X.; Huang, L.; Yang, M.; Chen, S. Identification and quantitative analysis of nucleosides and nucleobases in aqueous extracts of *Fritillaria cirrhosa* D. Don. using HPLC-DAD and HPLC-ESI-MS. *Anal. Lett.* **2011**, *44*, 2491–2502.

28. Zhao, F.; Qiu, X.; Ye, N.; Qian, J.; Wang, D.; Zhou, P.; Chen, M. Hydrophilic interaction liquid chromatography coupled with quadrupole-orbitrap ultra high resolution mass spectrometry to quantitate nucleobases, nucleosides, and nucleotides during white tea withering process. *Food Chem.* **2018**, *266*, 343–349.

29. Chang, X.; Zhang, Z.; Yan, H.; Su, S.; Wei, D.; Guo, S.; Shang, E.; Sun, X.; Gu, S.; Duan, J. Discovery of quality markers of nucleobases, nucleosides, nucleotides and amino acids for Chrysanthemi Flos from different geographical origins using uplc-ms/ms combined with multivariate statistical analysis. *Front. Chem.* **2021**, *9*, 689254.

30. Chang, X.; Wei, D.; Su, S.; Guo, S.; Qian, S.; Yan, H.; Zhao Ming, Z.; Erxin, S.; Dawei, Q.; Xiaodong, S.; et al. An integrated strategy for rapid discovery and prediction of nucleobases, nucleosides and amino acids as quality markers in different flowering stages of Flos Chrysanthemi using UPLC-MS/MS and FT-NIR coupled with multivariate statistical analysis. *Microchem. J.* **2020**, *153*, 104500.

32. Zhang, L.L.; Bai, Y.L.; Shu, S.L.; Qian, D.W.; Ou-yang, Z.; Liu, L.; Duan, J.A. Simultaneous quantitation of nucleosides, nucleobases, amino acids, and alkaloids in mulberry leaf by ultra high performance liquid chromatography with triple quadrupole tandem mass spectrometry. *J. Sep. Sci.* **2014**, *37*, 1265–1275

34. Wang, D.; Shi, L.; Fan, X.; Lou, H.; Li, W.; Li, Y.; Ren, D.; Yi, L. Development and validation of an efficient HILIC-QQQ-MS/MS method for quantitative and comparative profiling of 45 hydrophilic compounds in four types of tea (*Camellia sentences). Food Chem.* **2022**, *371*, 131201.

35. Wang, D.; Yu, Z.; Guo, J.; Liu, M.; Guan, M.; Gu, Y.; Siyu Li, S.; Ren, D.; Yi, L. Development and comparison of parallel reaction monitoring and data-independent acquisition methods for quantitative analysis of hydrophilic compounds in white tea. *J. Chromatogr. A* **2024**, *1715*, 464601.

36. Wang, D.; Li, Q.; Liu, R.; Xu, H.; Yin, Y.; Wang, Y.; Wang, H.; Bi, K. Quality control of *Semen Ziziphi Spinosae* standard decoction based on determination of multi-components using TOF-MS/MS and UPLC-PDA technology. *J. Pharm. Anal.* **2019**, *9*, 406–413.

38. Bai, X.; Zhou, L.; Wu, Y.; Xie, L. Alterations in the phytochemical composition and antioxidant activity of *Ligustrum robustum* according to continuous wet- and dry-heat treatment. *J. Food Sci.* **2023**, *88*, 1890–1904.

39. Ge, Y.; Li, N.; Fu, Y.; Yu, X.; Xiao, Y.; Tang, Z.; Xiao, J.; Wu, J.L.; Jiang, Z.H. Deciphering superior quality of Pu-erh tea from thousands of years' old trees based on the chemical profile. *Food Chem.* **2012**, *358*, 129602.

41. Fraser, K.; Lane, G.A.; Otter, D.E.; Harrison, S.J.; Quek, S.Y.; Hemar, Y.; Rasmussen, S. Non-targeted analysis by LC-MS of major metabolite changes during the oolong tea manufacturing in New Zealand. *Food Chem.* **2014**, *151*, 394–403.

42. Hong, C.; Yue, W.; Shen, Q.; Wang, W.; Meng, H.; Guo, Y.; Xu, W.; Guo, Y. Widely targeted metabolomics analysis reveals great changes in nonvolatile metabolites of oolong teas during Long-term storage. *Molecules* **2021**, *26*, 7278.

46. Long, J.; Chen, C.; Wang, Y.; Deng, H.; Zhang, Q.; Huang, L.; Xia, N.; Teng, J.; Zhu, P. Exploring the microbial community, physicochemical properties, metabolic characteristics, and pathways during tank fermentation of Liupao tea. *LWT* **2024**, *204*, 116449.

47. Wang, H.; Teng, J.; Huang, L.; Wei, B.; Xia, N. Determination of the variations in the metabolic profile and sensory quality of Liupao tea during fermentation through UHPLC-HR-MS metabolomics. *Food Chem.* **2023**, *404*, 134773.

48. Dong, H.; Li, Y.; Lai, X.; Hao, M.; Sun, L.; Li, Q.; Chen, R.; Li, Q.; Sun, S.; Wang, B.; et al. Effects of fermentation duration on the flavour quality of large leaf black tea based on metabolomics. *Food Chem.* **2024**, *444*, 138680.

49. Hua, J.; Ouyang, W.; Zhu, X.; Wang, J.; Yu, Y.; Chen, M.; Yang, L.; Yuan, H.; Jiang, Y. Objective quantification technique and widely targeted metabolomic reveal the effect of drying temperature on sensory attributes and related non-volatile metabolites of black tea. *Food Chem.* **2024**, *439*, 138154.

50. Shu, Z.; Ji, Q.; He, T.; Zhou, D.; Zheng, S.; Zhou, H.; He, W. Combined metabolome and transcriptome analyses reveal that growing under Red shade affects secondary metabolite content in Huangjinya green tea. *Front. Genet.* **2024**, *15*, 1365243.

51. Li, C.; Lin, J.; Hu, Q.; Sun, Y.; Wu, L. An integrated metabolomic and transcriptomic analysis reveals the dynamic changes of key metabolites and flavor formation over Tieguanyin oolong tea production. *Food Chem. X* **2023**, *20*, 100952.

52. Xue, J.; Liu, P.; Feng, L.; Zheng, L.; Gui, A.; Wang, X.; Wang, S.; Ye, F.; Teng, J.; Gao, S.; et al. Insights into the effects of fixation methods on the sensory quality of straight-shaped green tea and dynamic changes of key taste metabolites by widely targeted metabolomic analysis. *Food Chem. X* **2023**, *20*, 100943.

53. Xue, J.; Liu, P.; Guo, G.; Wang, W.; Zhang, J.; Wang, W.; Le, T.; Yin, J.; Ni, D.; Jiang, H. Profiling of dynamic changes in non-volatile metabolites of shaken black tea during the manufacturing process using targeted and non-targeted metabolomics analysis. *LWT* **2022**, *156*, 113010.

54. Fan, F.Y.; Huang, C.S.; Tong, Y.L.; Guo, H.W.; Zhou, S.J.; Ye, J.H.; Gong, S.Y. Widely targeted metabolomics analysis of white peony teas with different storage time and association with sensory attributes. *Food Chem.* **2021**, *362*, 130257.

55. Jia, W.; Shi, Q.; Shi, L.; Qin, J.; Chang, J.; Chu, X. A strategy of untargeted foodomics profiling for dynamic changes during Fu brick tea fermentation using ultrahigh-performance liquid chromatography-high resolution mass spectrometry. *J. Chromatogr. A* **2020**, *1618*, 460900.

58. Jon, C.S.; Yang, L.; Wang, Z.; Cui, M.; Sun, H.; Wang, L.; Liu, L.; Nardiello, D.; Quinto, M.; He, M.; et al. On the use of a 2D-carbon microfiber fractionation system to improve flow-injection QTOF-HRMS analysis in complex matrices: The case of *Abelmoschus manihot* flower extracts. *Analyst* **2022**, *147*, 819–827.

59. Fu, Y.; Shan, M.; Hu, M.; Jiang, Y.; Chen, P.; Chi, Y.; Yu, S.; Zhang, L.; Wu, Q.; Zhang, F.; et al. Chemical profiling of Banxia-Baizhu-Tianma decoction by ultra-fast liquid chromatography with tandem mass spectrometry. *J. Pharmac. Biomed. Anal.* **2019**, *174*, 595–607.

62. Luo, Y.; Yang, Y.; Yang, X.; Sun, C.; Chen, H. Quality evaluation of *Tetrastigma hemsleyanum* different parts based on quantitative analysis of 42 bioactive constituents combined with multivariate statistical analysis. *Phytochem. Anal.* **2022**, *33*, 754–765.

63. Liu, J.J.; Liu, Z.P.; Zhang, X.F.; Si, J.P. Effects of various processing methods on the metabolic profile and antioxidant activity of *Dendrobium catenatum* Lindley leaves. *Metabolites* **2021**, *11*, 351.

64. Ye, P.; Lu, J.; Li, M.; Zhang, H.; Chen, Y.; Wei, F. Comprehensive analysis of the compound profiles of Folium Camelliae Nitidissimae extract by ultrafast liquid chromatography with quadrupole-time-of-flight mass spectrometry and hepatoprotective effect against CCl_4-induced liver injury in mice. *Biomed. Chromatogr.* **2020**, *34*, e4817.

66. Chen, S.; Liu, J.Q.; Xiao, H.; Zhang, J.; Liu, A. Simultaneous Qualitative assessment and quantitative analysis of metabolites (phenolics, nucleosides and amino acids) from the roots of fresh *Gastrodia elata* using UPLC-ESI-Triple quadrupole ion MS and ESI-linear ion trap high-resolution MS. *PLoS ONE* **2016**, *11*, e0150647.

67. Li, Y.; Chen, N.; Li, W.; Lou, H.; Li, Y.; Xiong, Q.; Bai, R.; Wang, J.; Hu, Y.; Ren, D.; et al. Chemical profiling of ancient bud black tea with a focus on the effects of shoot maturity and fermentation by UHPLC-HRMS. *Eur. Food Res. Technol.* **2022**, *248*, 1379–1393.

68. Tan, J.; Dai, W.; Lu, M.; Lv, H.; Guo, L.; Zhang, Y.; Yin Zhu, Y.; Peng, Q.; Lin, Z. Study of the dynamic changes in the non-volatile chemical constituents of black tea during fermentation processing by a non-targeted metabolomics approach. *Food Res. Int.* **2016**, *79*, 106–113.

69. Yang, Z.; Kobayashi, E.; Katsuno, T.; Asanuma, T.; Fujimori, T.; Ishikawa, T.; Tomomura, M.; Mochizuki, K.; Watase, T.; Nakamura, Y.; et al. Characterisation of volatile and non-volatile metabolites in etiolated leaves of tea (*Camellia sinensis*) plants in the dark. *Food Chem.* **2012**, *135*, 2268–2276.

70. Xiang, Z.; Wang, Y.; Liu, S. The chemical and metabolite profiles of Gualou-Xiebai-Banxia decoction, a classical traditional Chinese medicine formula, by using high-performance liquid chromatography coupled with quadrupole time-of-flight mass spectrometry and in-house software. *J. Ethnopharmacol.* **2022**, *288*, 114994.

71. Zhou, J.; Yu, S.; Wang, B.; Wei, X.; Zhang, L.; Shan, M. Chemical profiling and quantification of Yihuang decoction by high performance liquid chromatography coupled with quadrupole time-of-flight mass spectrometry and a diode array detector. *J. Pharmac. Biomed. Anal.* **2023**, *224*, 115199.

72. Cui, H.; Mao, Y.; Zhao, Y.; Huang, H.; Yin, J.; Yu, J.; Zhang, J. Comparative metabolomics study of four kinds of Xihu Longjing tea based on machine fixing and manual fixing methods. *Foods* **2023**, *12*, 4486.

73. Sun, L.; Wen, S.; Zhang, S.; Li, Q.; Cao, J.; Chen, R.; Chen, Z.; Zhang, Z.; Li, Z.; Li, Q.; et al. Study on flavor quality formation in green and yellow tea processing by means of UPLC-MS approach. *Food Chem. X* **2024**, *22*, 101342.

74. Liu, X.; Tian, J.; Liu, G.; Sun, L. Multi-omics analysis reveals mechanisms of strong phosphorus adaptation in tea plant roots. *Int. J. Mol. Sci.* **2013**, *24*, 12431.

75. Deng, X.; He, S.; Han, Y.; Chen, Y. Metabolites profiling reveals the dynamic changes of non-volatiles in Pu-erh during Ganpu tea processing. *Food Chem. X* **2023**, *19*, 100774.

76. Liao, S.Y.; Zhao, Y.Q.; Jia, W.B.; Niu, L.; Bouphun, T.; Li, P.W.; Chen, S.-X.; Chen, W.; Tang, D.-D.; Zhao, Y.-L.; et al. Untargeted metabolomics and quantification analysis reveal the shift of chemical constituents between instant dark teas individually liquid-state fermented by *Aspergillus cristatus*, *Aspergillus niger*, and *Aspergillus tubingensis*. *Front. Microbiol.* **2023**, *14*, 1124546.

77. Xie, Y.; Zheng, J.Y.; Hou, Y.J.; Li, J.H.; Liu, W.H.; Sun, X.; Huang, Y.P. Hot-air full drying driven metabolome changes in white tea (*Camellia sinensis* L.). *Int. J. Food Prop.* **2023**, *26*, 2742–2756.

79. Huang, Y.; Liu, H.; Zhang, X.; Wu, Y.; Liu, Z.; Pang, Y.; Liu, R.; Yang, C.; Nie, J. Impact of storage time on non-volatile metabolites and fungal communities in Liupao tea using LC-MS based non-targeted metabolomics and high-throughput sequencing. *Food Res. Int.* **2023**, *174*, 113615.

80. Zhang, S.; Shan, X.; Niu, L.; Chen, L.; Wang, J.; Zhou, Q.; Yuan, H.; Li, J.; Wu, T. The integration of metabolomics, electronic tongue, and chromatic difference reveals the correlations between the critical compounds and flavor characteristics of two grades of high-quality Dianhong congou black tea. *Metabolites* **2023**, *13*, 864.

81. Yin, S.; Mei, Y.; Wei, L.; Zou, L.; Cai, Z.; Wu, N.; Yuan, J.; Liu, X.; Ge, H.; Wang, D.; et al. Comparison of multiple bioactive constituents in the corolla and other parts of *Abelmoschus manihot*. *Molecules* **2021**, *26*, 1864.

82. Wang, H.; Hua, J.; Yu, Q.; Li, J.; Wang, J.; Deng, Y.; Yuan, H.; Jiang, Y. Widely targeted metabolomic analysis reveals dynamic changes in non-volatile and volatile metabolites during green tea processing. *Food Chem.* **2021**, *363*, 130131.
